# Supportive Care Needs in Chinese, Vietnamese, and Korean Americans With Metastatic Cancer: Mixed Methods Protocol for the DAWN Study

**DOI:** 10.2196/50032

**Published:** 2024-04-22

**Authors:** Jacqueline H J Kim, Marjorie Kagawa Singer, Lisa Bang, Amy Ko, Becky Nguyen, Sandy Chen Stokes, Qian Lu, Annette L Stanton

**Affiliations:** 1 Department of Medicine University of California, Irvine Irvine, CA United States; 2 Department of Public Health University of California, Los Angeles Los Angeles, CA United States; 3 Vietnamese American Cancer Foundation Fountain Valley, CA United States; 4 Chinese American Coalition for Compassionate Care Cupertino, CA United States; 5 Department of Health Disparities Research The University of Texas MD Anderson Cancer Center Houston, TX United States; 6 Department of Psychology University of California, Los Angeles Los Angeles, CA United States

**Keywords:** Asian American, disparities, metastatic cancer, psychosocial, supportive care, unmet needs, well-being

## Abstract

**Background:**

Asian Americans with metastatic cancer are an understudied population. The Describing Asian American Well-Being and Needs in Cancer (DAWN) Study was designed to understand the supportive care needs of Chinese-, Vietnamese-, and Korean-descent (CVK) patients with metastatic cancer.

**Objective:**

This study aims to present the DAWN Study protocol involving a primarily qualitative, convergent, mixed methods study from multiple perspectives (patients or survivors, caregivers, and health care professionals).

**Methods:**

CVK Americans diagnosed with solid-tumor metastatic cancer and their caregivers were recruited nationwide through various means (registries, community outreach newsletters, newspapers, radio advertisements, etc). Potentially eligible individuals were screened and consented on the web or through a phone interview. The study survey and interview for patients or survivors and caregivers were provided in English, traditional/simplified Chinese and Cantonese/Mandarin, Vietnamese, and Korean, and examined factors related to facing metastatic cancer, including quality of life, cultural values, coping, and cancer-related symptoms. Community-based organizations assisted in recruiting participants, developing and translating study materials, and connecting the team to individuals for conducting interviews in Asian languages. Health care professionals who have experience working with CVK patients or survivors with metastatic solid cancer were recruited through referrals from the DAWN Study community advisory board and were interviewed to understand unmet supportive care needs.

**Results:**

Recruitment began in November 2020; data collection was completed in October 2022. A total of 66 patients or survivors, 13 caregivers, and 15 health care professionals completed all portions of the study. We completed data management in December 2023 and will submit results for patients or survivors and caregivers to publication outlets in 2024.

**Conclusions:**

Future findings related to this protocol will describe and understand the supportive care needs of CVK patients or survivors with metastatic cancer and will help develop culturally appropriate psychosocial interventions that target known predictors of unmet supportive care needs in Chinese, Vietnamese, and Korean Americans with metastatic cancer.

**International Registered Report Identifier (IRRID):**

DERR1-10.2196/50032

## Introduction

### Overview

Cancer is the leading cause of death for Asian Americans, the fastest-growing US immigrant group projected to outnumber Hispanic or Latinx immigrants by 2065 [1]. However, only 0.17% of the National Institutes of Health (NIH)–funded clinical research studies have involved Asian American, Native Hawaiian, and Pacific Islander participants (1992-2018) [2], and other foundational funding sources show a similar disparity for Asian Americans [3]. Of the many mental and physical health topics requiring further investigation [3], metastatic cancer survivorship is a significant priority given that Asian Americans are often diagnosed with a distant stage of cancer [4] such as Chinese Americans and Korean Americans with later staged colorectal cancer [5,6]. While progress in treatments and earlier detection have improved cancer survival rates overall [7], metastatic disease remains the major cause of cancer mortality [8]. The provision of quality supportive care is needed for Asian Americans with metastatic cancer, yet very little is known about the needs and preferences of this population. While the National Cancer Institute has funded cancer survivorship science since 1996, a 2020 analysis of NIH-funded research in advanced or metastatic cancer survivorship reported no studies of Asian Americans with metastatic cancer, and past survivorship research typically focused on survivors without detectable disease or active treatment [9,10]. Moreover, Asian-heritage cancer survivors are more likely to experience care-quality disparities [11], with those who are foreign-born especially likely to report unmet supportive needs after controlling for demographic and socioeconomic factors, health system and health care access, and comorbidities [12].

Psychosocial care needs are particularly important to evaluate, as the highest level of psychosocial distress is found in patients with advanced stages of disease [13]. These include anxiety, depression, death anxiety, demoralization, and a perceived inability to cope effectively, which can result in poor quality of life and premature mortality [14,15]. Patients with metastatic cancer have different needs, goals, and physical and psychosocial symptoms compared to patients with earlier-stage cancer, with individuals with metastatic cancer facing greater uncertainty about their prognosis and fear of disease progression [10,16,17]. Evidence suggests early supportive care needs (ie, after diagnosis in active treatment) should also be examined, as it can result in better quality of life and a reduction in functionally impairing symptoms [18].

Chinese-, Vietnamese-, and Korean-descent (CVK) Americans experience greater cancer-specific mortality in contrast to other Asian ethnic groups [19-22] and likely experience difficulties accessing high-quality care due to 46%-60% reporting limited English proficiency [23]. Very little is known about CVK Americans’ metastatic cancer survivorship and how similarities in philosophical traditions, values, and norms may guide distinct views about what is optimal for supportive care in metastatic cancer (eg, Confucian-heritage–based interdependent norms for East and Southeast Asian ethnic groups [24,25]). For example, Confucian-heritage individuals’ self-concept often includes close others [26,27] and CVK patients’ supportive care needs may be influenced by social harmony-based expectations such as self-sacrifice amid profound stress [28,29] or preferring indirect support [30] to minimize direct discussion of stigmatizing subjects such as cancer [31-33]. Supportive interventions or resources that allow for a reduction in directly verbalizing distress may be preferred (eg, writing [34] or use of web-based or social chat platforms [35]). As such, this generative study aimed to produce an initial glance at the supportive care needs and preferences of CVK patients or survivors. Multiple perspectives were sought as cancer survivorship care is coordinated across multiple levels, including close interpersonal relationships, health care organizational settings, and community settings [36]. This protocol outlines the multilevel assessment undertaken through multiple informants and study components.

### Objectives

This study aims to present an embedded mixed methods study protocol for examining the supportive care needs and preferences of CVK patients or survivors with metastatic cancer through multiple informants (patients, caregivers, and health care professionals). To our knowledge, this is the first multiperspective study focused on Asian American metastatic cancer survivorship. This protocol will allow for an understanding of the components of the study, and findings from this study will refer to this protocol to showcase how community-engaged collaboration aided the development of culturally relevant, adapted psychosocial interventions or resources targeting known predictors of continued unmet supportive care needs.

## Methods

### Overview

**Figure 1:**
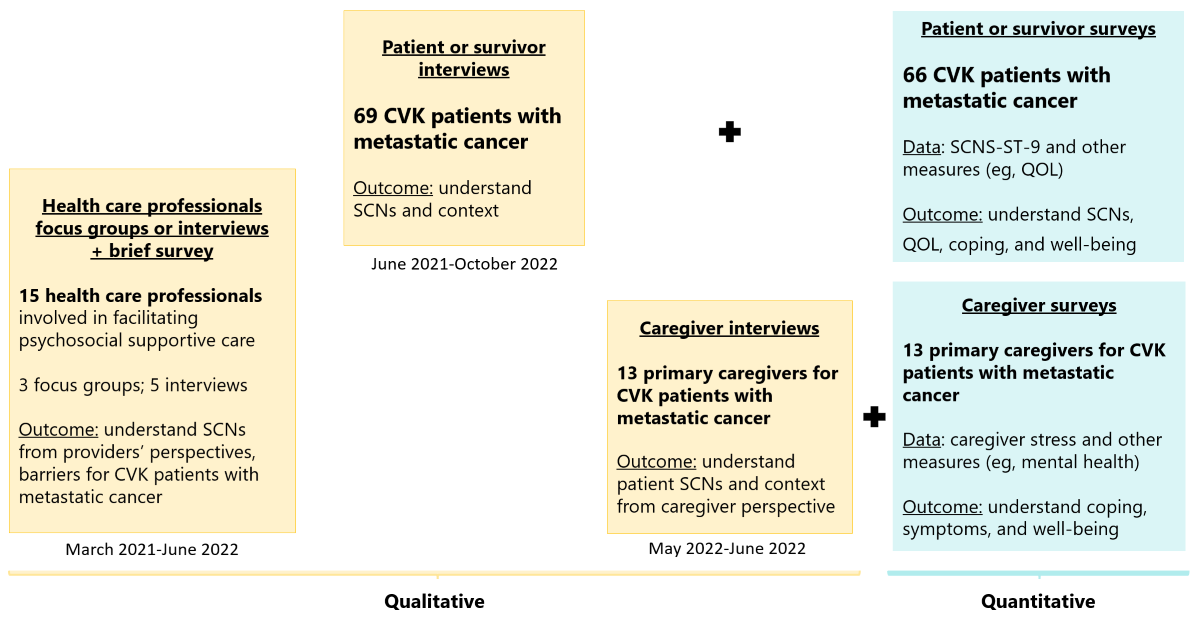
Convergent mixed methods design and timeline. CVK: Chinese-, Vietnamese-, and Korean-descent; QOL: quality of life; SCN: supportive care need; SCNS-ST-9: Supportive Care Needs Survey-Screening Tool.

We conducted a qualitative (QUAL) and quantitative (quan) convergent mixed methods study on supportive care needs with CVK survivors living in the United States and diagnosed with solid-tumor metastatic cancer (Figure 1). A convergent mixed methods design was selected to provide a rich description of supportive care needs, including any similarities or discrepancies in self-reporting through interview or survey assessment. The strength of a convergent design is its ability to collect both qualitative and quantitative data simultaneously and promptly, which fits well with the generative “snapshot” purpose of this study given the lack of knowledge about conducting research with this understudied population diagnosed with stage IV cancer. With guidance from a qualitative expert (coauthor MKS), the qualitative portion was prioritized to engage an understudied population of cancer survivors who may face cancer-related stigma due to cultural beliefs about cancer. We invited survivors’ caregivers to provide their perspective on survivors’ supportive care needs through interviews. Health care professionals’ perspectives on the supportive care needs of CVK metastatic cancer patients or survivors and their families were also examined qualitatively. All study materials were matched to participants’ language preferences in English, traditional Chinese, simplified Chinese, Vietnamese, and Korean.

### Participants

The study population included CVK individuals with solid-tumor metastatic cancer and caregivers (aged ≥18 years) living in the United States. Patients or survivors and caregivers were primarily recruited through partnered community outreach and supplemented by the University of California, San Francisco CARE (Collaborative Approach for Asian Americans, Native Hawaiians, and Pacific Islanders Research and Education) Registry and the University of California, Los Angeles (UCLA) Cancer Registry. Trained bilingual research assistants who speak English and Chinese (Mandarin or Cantonese), Vietnamese, or Korean called potential patient or survivor and caregiver participants to introduce and explain the study, screen for eligibility, and obtain consent for participation. Patient or survivor and caregiver participants received a US $90 e–gift card upon completing all parts of the study (brief demographic questions, interview, and survey). Patient or survivor participants who started the study but were unable to complete it, or declined to further participate for health reasons were also sent US $90 e–gift cards for their efforts. Health care professionals received a US $100 e–gift card upon completing the study.

### Eligibility Criteria

The inclusion criteria for patients or survivors were (1) individuals diagnosed with de novo or recurrent, stage IV, solid tumor metastatic cancer; (2) self-identifying as Chinese, Vietnamese, or Korean and fluent in English, Korean, Cantonese, Mandarin, or Vietnamese; (3) aged 18 years or older; and (4) living in the United States. The inclusion criteria for caregivers were (1) individuals who self-identified or were patient- or survivor-identified as the primary caregiver (a close relative, friend, or partner who is doing most of the work to help take care of daily activities or medical care) for a person diagnosed with de novo or recurrent solid tumor metastatic cancer; (2) aged 18 years or older; and (3) living in the United States. If any patient or survivor or caregiver reported a diagnosis of schizophrenia or bipolar disorder, post hoc exclusion was to be considered based on any evidence of cognitive impairment presented to the research team during interactions or interviews. Health care professionals were eligible if they (1) were involved in facilitating patients’ connection to or providing supportive care; (2) have been practicing at their medical or health center-related organization for 6 months or more; and (3) regularly worked with or have worked with at least 2 Chinese, Vietnamese, or Korean American patients with metastatic cancer.

### Recruitment

Patient or survivor participants were initially recruited from the Greater Los Angeles Area and Orange County in Southern California. Recruitment was expanded nationwide due to limitations in reach and access resulting from the study being conducted during the height of the COVID-19 pandemic. Advertising and community outreach were focused on locations with a greater presence of Chinese, Vietnamese, and Korean communities (Los Angeles-San Gabriel Valley region, San Francisco Bay Area region, Buena Park, La Palma, Little Saigon area in Orange County, Irvine, Houston, Seattle, New York, etc). A majority of participants were recruited through flyers and community outreach (community organizations, churches, temples, libraries, community centers, senior centers, and ethnic supermarkets), physician referrals, doctor’s offices, hospices, coverage on ethnic television, radio, or newspapers, social media (Facebook and Instagram), and word-of-mouth. Partner community organizations (Chinese American Coalition for Compassionate Care [CACCC] 美華慈心關懷聯盟 and the Vietnamese American Cancer Foundation [VACF] Hội Ung Thư Việt Mỹ) helped increase word-of-mouth recruitment by using their websites, social media, and radio broadcasts to publicize the research. VACF lay navigators actively distributed study flyers with their community during the organization’s events, such as the drive-by COVID-19 relief resource distribution. Participants were also later recruited through the University of California, San Francisco CARE Registry and the UCLA Cancer Registry when open to non-COVID-19 research. Recruitment efforts for caregivers began with paired dyads for more focused qualitative synthesis; thus, we first recruited through patient or survivor participants who identified their primary caregivers and gave assent for contact. Following this, the research team sought to recruit caregivers unpaired with patient or survivor study participants.

Health care professionals were recruited through the Describing Asian American Well-Being and Needs in Cancer (DAWN) Study community advisory board members’ referrals of those who may best understand the supportive care needs of CVK adults with metastatic cancer, based mostly in Southern California.

### Analysis Plan

The patient or survivor and caregiver interviews were transcribed in English, Mandarin, Cantonese, Korean, and Vietnamese. Asian language transcripts were translated, and English transcripts will be analyzed. The health care professional interviews and focus groups were transcribed in English. All transcription and translation work was reviewed and corrected by the multilingual research team. All qualitative data will be examined for thematic content with reflexive thematic analysis [37,38]. Reflexive thematic analysis is a theoretically flexible approach that allows for the presentation of shared meaning in data by emphasizing researcher reflexivity as a key process in the analysis and acknowledging that the findings are embedded in this context [37,38]. Self-reflexivity will be emphasized before and throughout the analysis and interpretation. Coders and the principal investigator (JHJK) will discuss the influence of their backgrounds and preconceptions and keep memos for a record of analysis and interpretations. As the lead investigator (JHJK) approaches research with a critical realist framework and is a clinical psychologist with knowledge of mental health service use disparities, cultural variations in symptomatology and coping, and stressors faced by immigrant families, findings are likely to be influenced by this perspective.

The research team will first familiarize themselves with the data through independent reading of the interview transcripts and generate initial codes and candidate themes to engage in reflexive thematic analysis. Inductive “bottom-up” coding will be completed first for a full description of participants’ perspectives and experiences. Then, deductive “top-down” coding will be performed centered on the research question and any relevant supportive oncology concepts of additional significance. Themes will be reviewed and refined to best represent participant perspectives. Seeking saturation and minimum sample sizes is not compatible with reflexive thematic analysis, and our sampling was pragmatic and acceptable within reflexive thematic analysis (ie, the research team sought to conduct as many qualitative data collections as possible during the project period). Self-reported supportive care needs, demographics, and medical variables will also be summarized with univariate statistics. Chi-square tests will examine any differences in supportive care needs (eg, US- or foreign-born). Joint display results will be produced for the supportive care needs described by CVK patients or survivors. For the health care professionals’ parallel-format focus group and interview data, candidate themes were compared across the focus group data and interview data to arrive at final themes.

### Data Management and Confidentiality

When participants enrolled in the study, they were assigned a random study ID number to label self-report and interview data. Self-report and interview data were further deidentified. Any potentially identifying records are kept password-protected on a secure server. All analyses will be reported as aggregate data or in an anonymized format for qualitative excerpts.

### Data Collection Overview

#### Patients or Survivors and Caregivers

After obtaining consent, self-reported demographic information (eg, age, sex, ethnicity, education level, income, length of US residency, language use, religion, employment status, insurance status, and subjective social status captured using the community version of the MacArthur Scale of Subjective Social Status [39]), acculturation level (from the Brief Acculturation Scale for Hispanics [40]), and cancer history (eg, type of metastatic cancer, diagnosis date, and treatments) were collected by telephone immediately following the screening call or at another scheduled time. Following the demographic and cancer history questions call, participants were scheduled for a 1-1.5-hour interview over telephone or videoconference through Zoom (Zoom Video Communications). When scheduling and sending reminders for the interview, participants were asked to secure a private location where they could speak freely and confidentially, in addition to a suggestion to use headphones for added privacy. Participants’ privacy and comfort with their interview location were confirmed before beginning the interview. In the rare circumstance that the participant needed another location for continued privacy for the interview, participants were offered a break period or to reschedule to continue the interview in a confidential manner. After the interview, participants completed a 30-minute survey on paper or a web-based survey within 1 week of the interview. All participants were encouraged to complete their survey on the same day as the interview, in one sitting, if possible, within 1 week from the interview date. They were reminded to complete the survey through their preferred method of communication. Participants with lower literacy were supported by trained bilingual research assistants over the phone. Caregivers underwent the same study process as patients or survivors (brief demographic questions through phone, 1-hour interview, and 30-minute survey).

#### Health Care Professionals

Health care professionals were provided study information sheets with the opportunity to ask questions about eligibility before scheduling the 1-hour focus groups or interviews led in English by the principal investigator. Focus groups and parallel key informant interviews took place over Zoom. Focus groups were composed of a minimum of 3 health care professionals to allow for greater depth of responses from each participant attending the focus group, as well as to accommodate scheduling restrictions imposed by their COVID-19 workload. The three focus group compositions were balanced out as much as possible: (1) social worker, bilingual radiation oncologist, and bilingual medical oncologist; (2) social worker, bilingual medical oncologist, and medical oncologist; and (3) chaplain, bilingual hematology oncologist, hematology oncologist, and bilingual medical oncologist. Following the focus groups, parallel-format key informant interviews were the only feasible way to gain additional perspectives due to time limitations from health care professionals’ COVID-19 workload—this alternate participation option was pursued when health care professionals could not otherwise participate in the focus groups. In reminders before the interview, participants were asked to secure a private location where they could speak freely and confidentially and were suggested to use headphones for added privacy. Before beginning each focus group or interview, health care professionals confirmed their eligibility and provided oral or web-based survey consent. They also confirmed privacy and comfort with their location of remote participation. The demographics of health care professionals were collected before the interviews through a brief demographic questionnaire on the web. Self-reported demographic information includes age; profession; sex; ethnicity; years since clinical licensure; years since obtaining an MD degree; hospital or clinic affiliation; months employed at the institution, hospital, or clinic; if bilingual in Chinese, Vietnamese, or Korean and if using the endorsed language in clinical practice; years working with patients with metastatic cancer; the number of CVK patients with metastatic cancer seen in the past month and per month before COVID-19; and level of comfort caring for CVK patients with metastatic cancer.

### Semistructured Guides for Qualitative Data

#### Patients or Survivors and Caregivers

All interviews were conducted using semistructured topic guides. Interviews were conducted in English, Chinese (Cantonese or Mandarin), Vietnamese, and Korean by language-matched, trained interviewers and the principal investigator. Interviews lasted 60-90 minutes for both the patients or survivors and their caregivers. Interviews took place through Zoom or telephone calls and were audio-recorded with participants’ permission. The main research question domains for patients or survivors and caregivers were (1) cultural influence on coping, needs, and preferences; (2) cultural fit and adequacy of existing or offered supportive care after diagnosis; (3) unmet supportive care needs (physical vs emotional priorities); and (4) psychosocial intervention or resource preferences (modality and timing). Supportive care needs explored included informational, patient care support, daily living, physical, psychological, or emotional, as well as how people’s social lives (interpersonal relationships, marriage, sexuality, and spirituality) were affected. Caregivers were asked to provide information regarding their own experiences as well as the patient’s or survivor’s experiences in these domains and their insights about patients’ or survivors’ needs. Caregivers were also asked about how their relationship with the patient or survivor had changed or stayed the same if they were married or in a committed relationship. In the fourth domain, participants were asked to rank potential psychosocial interventions based on perceived helpfulness for patients or survivors with metastatic cancer of their ethnicity: writing privately, a website or blog for close family or friends, social media with close family or friends, peer education or support, and professionally trained counseling. Images representing each type of intervention were provided for participants to reference during the interview. For interviews in which participants mentioned end-of-life or openness about mortality, and time permitting, patients or survivors were shown an image of CACCC’s Heart to Heart Café cards to ask for their thoughts regarding a culturally based intervention for end-of-life discussions with family or friends. This last intervention image was presented in a tailored manner for each individual because of ethical concerns raised by community and scientific advisors given the known stigma and taboo related to the end of life in Asian cultures [31], the limited scope of the project, and the lack of culturally and linguistically matched supportive or counseling resources available to all participants.

#### Health Care Professionals

A semistructured interview guide was developed and used for the focus groups and interviews in English. All participants were asked the same questions per the interview guide by the same facilitator. Domains of inquiry included (1) any difference in care provided for CVK patients with metastatic cancer; (2) challenging scenarios for CVK patients with metastatic cancer; (3) health care professionals’ perceptions of the most important unmet supportive care needs, barriers to meeting those unmet needs, and potential solutions; and (4) the type of supportive care that would be most helpful and culturally appropriate. Focus group participants were scheduled for an individual follow-up interview if there were any comments to probe for additional clarifying information. Focus group participants’ thoughts were shared with parallel-format individual interview participants, as appropriate, to elicit their reactions and thoughts as would occur if they had participated in a focus group.

### Survey Measures for Quantitative Data

#### Overview

All measures for the study were provided in English, Chinese (traditional or simplified), Vietnamese, or Korean. If not already available or needing wording modification, simplified and traditional Chinese, Vietnamese, and Korean versions of measures were developed using a rigorous translation methodology. A total of 2 independent forward translations, 1 independent back translation, and reconciliations of translations were completed with feedback from community partner organization representatives and bilingual research team members from each language group. Feedback from the Chinese, Vietnamese, and Korean community organization representatives included identifying the best phrasings for the United States context while maintaining the meaning of the original English measure. If not in the public domain, permission was obtained from the measure authors.

#### Patients or Survivors

The primary survey for a convergent mixed methods examination of patients or survivors with metastatic cancer’s supportive care needs was measured by the Supportive Care Needs Survey-Screening Tool [41]. The Functional Assessment of Cancer Therapy General-7 measure was used to assess quality of life [42]. Participants’ other patient-reported outcomes were assessed with the National Comprehensive Cancer Network distress thermometer [43], an 8-item Patient Health Questionnaire Depression Scale for depression [44], Generalized Anxiety Disorder Scale for anxiety [45], the Multidimensional Fatigue Symptom Inventory-Short Form physical subscale for fatigue [46], the 6-item Impact of Event Scale for cancer-specific traumatic stress [47], the pain intensity, enjoyment of life, general activity 3-item scale for pain assessment [48], and the 2-item Sleep Condition Indicator for sleep disturbance [49]. Considering the importance of coping in determining quality of life and other patient-reported outcomes, coping was evaluated using the Brief Coping Orientation to Problems Experienced Inventory [50] and the Emotional Approach Coping Scales (emotional processing and emotional expression) [51]. The Mental Health Continuum Short Form examined well-being [52], and other resilience factors were examined with the Gratitude Questionnaire-6 [53], the mindfulness subscale of the Self-Compassion Scale [54], the preparedness subscale of the Quality of Life at the End of Life-Cancer measure [55], the UCLA 3-Item Loneliness Scale [56], and the Social Provisions Scale [57].

We also measured additional exploratory concepts related to cultural factors that may affect supportive care needs and quality of life among CVK adults with metastatic cancer. The Asian American Values Scale was modified as a brief 9-item pilot version to assess how much participants valued family collectivism, conformity to norms, and emotional self-control [58], alongside the harmony subscale of the Brief Collectivism Questionnaire [59]. Other culturally relevant concepts measured include ambivalence of emotional expression with the 4-item Ambivalence Over Emotional Expressiveness Questionnaire [60], internalized metastatic cancer-related stigma (modified Lung Cancer Stigma Inventory subscale) [61], self-perceived burden upon others [32,62], the patient’s level of sacrifice for their family using a modified Sacrifice for Close Others Scale [63], and a question about lifetime experience of personal or sociopolitical trauma. All measures were completed in one survey assessment after the interview.

#### Caregivers

Caregivers filled out the same surveys as patients or survivors for cancer-related coping, mental and physical health symptoms, resilience factors such as gratitude, and cultural values. Additionally, their caregiving relationship was measured with the 4-item Quality of Caregiver-Care Recipient Relationship [64,65] and caregiving stress was measured with the Caregiver Intrapsychic Stress and Strain Scales [66]. All measures were completed in one survey assessment after the interview.

### Partnered Community Involvement

The study was conducted in collaboration with community organizations that were cultural and community-based advisors for the research and supported all nonengaged aspects of the study such as prioritizing research questions, informing the design of the study including participant eligibility criteria, recommending culturally relevant concepts to assess (eg, stigma or burden), translating study materials, reviewing the ethics of the research procedures, supporting recruitment efforts, and recommending bilingual research assistant candidates for interviewing participants. The CACCC (美華慈心關懷聯盟) is a coalition in Cupertino, California that addresses issues related to serious illnesses in the Chinese American community and partnered for the Chinese portion of the study. The VACF (Hội Ung Thư Việt Mỹ) in Orange County, California is an organization that provides cancer education, resources, and services and is involved in research and advocacy for cancer prevention to improve quality of life and outcomes in the Vietnamese community and partnered for the Vietnamese portion of the study. The DAWN Study community advisory board comprising multidisciplinary health care professionals (psychology, oncology, palliative care, or public health), patient advocates, and community-based organizational representatives, also contributed to the development and conduct of the study through quarterly meetings. For example, the eligibility criteria for all participants were informed through community advisory board discussions, with the minimal length of employment for health care professionals set at 6 months for the health care professional to have had time to adjust to their health care system and be able to provide an “insider” perspective. The community advisory board included at least one bilingual representative with cultural knowledge relevant to CVK patients and their families.

### Ethical Considerations

Institutional review board approval was obtained from UCLA (20-001554) and the University of California, Irvine (1541;2391). Oral or web-based informed consent was obtained from all participants in this study. Nonregistry-based patient or survivor participants completed a web-based screening and consent form, or expressed interest through email or phone and oral consent was obtained over the phone by trained research assistants. Registry-based patient or survivor participants received a letter or email with the study information sheet and a postage-paid postcard to indicate their interest. Research team members followed up with all potentially eligible registry-based patients or survivors to complete the screening and oral consent by phone if participants had not completed the web-based screening and consent on their own. For all participants who consented through a web-based survey, we ensured informed consent by verbally reconfirming an understanding of the purpose of the study, what participation entails, and rights as participants at the beginning of the first portion of the study.

## Results

The study was funded for 2 years, from June 2020 to June 2022, by the NIH-National Cancer Institute with supplement funding from the UCLA Institute of American Cultures and Asian American Studies Center. Recruitment began in November 2020, and data collection was completed in October 2022. A total of 74 patients or survivors enrolled in the study from various US states (66 from California, 3 from Texas, 2 from Washington, 1 from Delaware, 1 from Nevada, and 1 from Connecticut); 69 patients or survivors completed the interview, and 66 patients or survivors completed both the interview and survey. A total of 13 caregivers enrolled in the study (12 from California and 1 from Washington) and completed all portions of the study. All 13 caregivers were linked with a patient or survivor participant in the study; no unlinked caregivers joined the study. A total of 15 health care professionals enrolled in the study, resulting in 3 focus groups and 5 parallel-format interviews. No post hoc exclusions were necessary. We completed the remaining data management in December 2023. A manuscript on health care professionals has been accepted, and manuscripts for convergent mixed method results for patients’ or survivors’ supportive care needs and caregiver perspectives will be submitted in 2024.

## Discussion

This study is one of the first to examine the unmet supportive care needs of Asian Americans with metastatic cancer through interviewing patients or survivors, caregivers, and health care professionals for a more comprehensive understanding, using culturally and linguistically appropriate study materials and community-based participatory research principles. The perspectives of interested parties, which include patients or survivors, caregivers, community members, and health care professionals, will help advocate for this understudied population and inform the next steps to develop, test, and disseminate supportive care resources and interventions that are culturally relevant. By studying adults from Confucian-heritage cultures, this study may uncover cultural factors to be considered in the provision of supportive care. However, given the focus on only CVK adults, the results of this study may not apply to other Asian ethnicities that do not share a Confucian heritage. This research design may be adaptable for other understudied Asian Americans with metastatic cancer to facilitate quality care and intervention development.

Strengths of this study include the involvement of the community through their active participation from the design of the study to the analysis, interpretation, and dissemination of results. Multidisciplinary community advisors who closely work with metastatic cancer survivors and community-based organizations shaped the study to be more culturally appropriate. All steps of the research were available in English, Chinese, Vietnamese, and Korean, and the study was designed to be flexible around participants’ schedules (including evenings and weekends) and remove the burden of transportation by being remote.

Challenges encountered in this study include technological and logistical difficulties in offering multiple modalities for participation. Considering that some participants had limited familiarity with technology such as Zoom and SMS text messaging and relied on telephone calls, it was challenging, at times, to connect with individuals to complete every part of the study. Another challenge faced was during the recruitment process, as some participants did not know the staging of their cancer. Diagnoses needed to be clarified through caregivers or with detailed follow-up questions about where the cancer had spread. Though every effort was made to ensure privacy, confidentiality, and independence in participant interviews, one patient strongly preferred that their caregiver be present. Given the patient’s physical supportive needs and sporadic difficulty with speech due to cancer, this patient’s decision was honored. An explanatory discussion of the research process ensured understanding from both the patient and caregiver that interview and survey responses should be the patient’s most direct and honest thoughts. We included this dyad in the study as they were otherwise eligible, and it was deemed the most ethical route per community advisors’ recommendations. Lastly, due to COVID-19, opportunities to establish community-based relationships in person were unavailable, and future research participation may be even more plentiful with such efforts. In particular, partnering with locally based community-based organizations and research registries outside of California will be key to reaching a broader US sample, as the majority of the participants in this study were from California.

## Abbreviations

CACCC: Chinese American Coalition for Compassionate Care
CVK: Chinese-, Vietnamese-, and Korean-descent
DAWN: Describing Asian American Well-Being and Needs in Cancer
NIH: National Institutes of Health
UCLA: University of California, Los Angeles
VACF: Vietnamese American Cancer Foundation

## Data Availability

The data sets generated during this study are not publicly available. The original consent for study participants did not state such an intention to share the data publicly with others outside of the research team, and some participants are no longer reachable for a reconsent procedure. Confidentiality of participants is of utmost priority, and upon review of the data, there is a possibility that, with known collaborators on this project, demographic characteristics, diagnostic information, interview response patterns, and details, in combination, could make participants recognizable to others in the smaller Asian American network serving metastatic cancer patients or survivors, to participants’ health care providers, or to participants’ colleagues. Therefore, only restricted data reviews may be discussed with the corresponding author upon reasonable request.
